# Evaluation of Skeletal Muscle Dysfunction Associated With Acute Inflammation by Electrical Impedance Myography: A Case Report on Skeletal Muscle Dysfunction After Cardiac Surgery and Literature Review

**DOI:** 10.7759/cureus.20166

**Published:** 2021-12-04

**Authors:** Hiroki Sato, Takao Nakamura

**Affiliations:** 1 Department of Radiological Technology, Graduate School of Health Sciences, Okayama University, Okayama, JPN; 2 Department of Rehabilitation Medicine, Kawasaki Medical School, Kurashiki, JPN; 3 Department of Rehabilitation Center, Kawasaki Medical School Hospital, Kurashiki, JPN; 4 Graduate School of Health Sciences, Okayama University, Okayama, JPN

**Keywords:** cardiac surgery, ultrasonography, muscle quality, phase angle, electrical impedance myography

## Abstract

Electrical impedance myography (EIM) is an evaluation technique for skeletal muscles that uses electrical impedance technology. Recent reviews have shown that EIM is useful as a method to assess changes in skeletal muscle quality and quantity with aging. These may be utilized for functional changes in inflammatory skeletal muscles, such as disease and operation. In this report, the impedance parameters using EIM present perioperative skeletal muscle changes in patients after cardiac surgery. In addition, we will describe the efficacy of EIM in skeletal muscle dysfunction due to inflammation or disease. This study aimed to elucidate the efficacy of EIM in acute inflammation-associated skeletal muscle dysfunction.

## Introduction

Bioelectrical impedance technology has been used in the medical field to assess body composition, such as body water, skeletal muscle, and fat content [1]. Recently, a diagnostic technique called electrical impedance myography (EIM) has been proposed, which measures the intrinsic impedance of skeletal muscle using these techniques [2]. In EIM, a variety of numerical values are calculated, including the phase angle (PA) and the ratio of intracellular to extracellular fluid resistance (*R_i_*/*R_e_*), the beta parameter (*β*) that represents tissue uniformity, and the center frequency (*f_c_*). These parameters have been shown to be related to muscle fiber mass and fascial function in skeletal muscles [2]. We investigated the effectiveness of skeletal muscle quality and quantitative assessment of the impedance parameters of the upper and lower limb measured by direct segmental multifrequency bioelectrical impedance analysis and found that *R_i_/R_e_* correlates with muscle mass and beta parameters with muscle mass indices and that each has an independent effect on muscle strength [3]. Thus, PA, which reflects the state of cells, may be applied to evaluate quality and quantity by separating them into their constituent components.

Skeletal muscle function is altered not only by aging but also by diseases and systemic inflammation [4]. Skeletal muscle dysfunction associated with systemic inflammation, especially after cardiac surgery, has been shown to cause a severe functional decline in a short period of time [5]. However, there is no standardized evaluation method immediately after surgery because general conditions and pain make conventional muscle strength evaluation difficult. Although EIM is likely to be effective for such changes in skeletal muscle function in the acute phase, no studies have investigated changes in skeletal muscle function in the acute phase using impedance parameters by EIM.

This case report presents perioperative changes in skeletal muscle in patients after cardiac surgery using impedance parameters by EIM. In addition, EIM insights into acute inflammation and disease-induced skeletal muscle dysfunction are discussed.

## Case presentation

A 56-year-old Japanese male with severe aortic regurgitation (maximum velocity of blood flow at the aortic valve opening, 2.3 m/s; aortic valve area, 1.54 cm^2^; ejection fraction, 36%) and dilatation of the ascending aorta (5.1 cm) on echocardiography was indicated for surgery. Concomitant diseases included hypertension, dyslipidemia, and diabetes mellitus (type II). He worked at his office and was independent in his daily life. Rehabilitation was initiated before surgery. Before surgery, physical function tests by the physiotherapist revealed a body weight of 72.0 kg, grip strength of 38.0 kg, leg extension strength of 138.0 Nm, skeletal muscle index (SMI) of 8.1 kg/m^2^, short physical performance battery (SPPB) of 12 points, and no abnormalities in physical function. Skeletal muscle function was evaluated using the rectus femoris muscle. Muscle thickness and intensity were measured using ultrasonography (SonoSite M-turbo, Fujifilm, Japan) [6]. EIM was measured using a bioelectrical impedance spectroscopy unit (SFB7, Impedimed, Australia) to measure PA and *β* (details of measurement and analysis methods are given in the Appendix).

Approximately one week of preoperative rehabilitation was followed by Bentall surgery (extracorporeal circulation time, 212 min; anesthesia time, 497 min; operation time, 408 min). Rehabilitation (e.g., early mobilization and respiratory rehabilitation) was started the day after surgery. On a postoperative day 1 (POD 1), the patient was weaned from a ventilator, and on POD 3, the patient was able to walk 100 m. There were no complications, the patient became independent in daily life on POD 7, and the patient was discharged from the hospital on POD 18. Physical function was evaluated in POD 14, and skeletal muscle function was evaluated preoperatively and in PODs 1, 3, 5, 7, and 14.

The physical function evaluation of POD 14 showed a body weight of 66.7 kg, grip strength of 36.5 kg, leg extensor strength of 126.0 Nm, SMI of 7.8 kg/m^2^, and SPPB of 12 points. Table 1 shows the evaluation of perioperative skeletal muscle function. In the evaluation of skeletal muscle function, ultrasonography showed a decrease in the thickness of the rectus femoris muscle from 1.81 cm preoperatively to 1.64 cm on POD 14. The increase in muscle intensity peaked on POD 5 and gradually improved after POD 7. In the EIM, PA decreased from 14.6° before surgery to 11.1° on POD 14. The *β* peaked at POD 3, and the POD 14 level improved to the same level as the preoperative level. Increased from baseline (preoperatively) to 0.32 and 0.36 on POD 5 and POD 14, respectively.

**Table 1:** Ultrasonography and EIM of the rectus femoris during perioperative US: ultrasonography, MT: muscle thickness, MI: muscle intensity, EIM: electrical impedance myography, PA: phase angle, β: beta parameter, Ri /Re: ratio of intracellular fluid resistance to extracellular fluid resistance, fc: central relaxation frequency, POD: postoperative day

	Preoperative	POD 1	POD 3	POD 5	POD 7	POD 14
US						
MT (cm)	1.81	1.60	1.71	1.65	1.65	1.64
MI	36.1	53.3	56.2	52.6	48.6	40.5
EIM						
PA (°)	14.6	14.0	13.5	12.7	13.0	11.1
β	0.793	0.748	0.722	0.761	0.773	0.797
R_i_/R_e_	0.32	0.31	0.31	0.34	0.34	0.36
f_c_ (kHz)	24.2	29.3	28.3	38.7	36.6	35.5

## Discussion

To the best of our knowledge, this is the first report to evaluate EIM for inflammatory skeletal muscle dysfunction after surgery. Previous studies have shown that the impedance parameters measured by EIM are associated with age-related changes in skeletal muscle and neuromuscular diseases [2]. In this case, acute changes in skeletal muscle function caused by inflammatory reactions after thoracotomy were serially evaluated using ultrasonography and EIM to determine the efficacy of EIM. In addition to the changes in skeletal muscle mass and quality measured using ultrasonography, PA decreased markedly. A noteworthy finding is that the changes in PA-constituted *β* and occurred independently and were more sensitive than the quantity and quality indices of skeletal muscle assessed with ultrasonography.

Skeletal muscle damage associated with acute inflammation is caused by infiltration of inflammatory cytokines, leading to a decrease in fascial function and an increase in non-contractible tissue within the muscle [4]. Changes in skeletal muscle quality due to these disorders cause marked muscle weakness in a short period of time. After cardiac surgery, muscle weakness of ≥10% during POD 7 is associated with inflammatory muscle protein catabolism [5]. As in the previous study, muscle weakness and a decrease in muscle mass of approximately 10% were observed in this case after the operation. Muscle intensity increased more at POD 1 and gradually improved after peaking at POD 3. Meanwhile, the EIM results showed that PA gradually decreased from 14.6° (preoperatively) to 13.0° (a decrease of 11.2%) and 11.1° (a decrease of 23.8%) on PODs 7 and 14, respectively. Increased preoperatively by 0.32 versus POD 14 by 0.36 (an increase of 13.0%); decreased at POD 3 and then improved at POD 14. These results also suggest that EIM parameters may be sensitive, responsive parameters, and effective for postoperative changes in skeletal muscle mass and quality.

EIM has been reported to be effective in aging and sarcopenia-induced changes in skeletal muscle function [2]. Because EIM is sensitive to minor morphological changes, such as cell density and size in tissues, it is useful not only for age-related changes but also for disease-genic changes [7]. Previous studies evaluating disease-specific changes have examined changes over time in disease progression in amyotrophic lateral sclerosis (ALS) and Duchenne muscular dystrophy (DMD) [8,9]. Shefner et al. [8] longitudinally evaluated EIM in patients with ALS and ALS-Minics (motor neuropathy, radiculopathy) and healthy subjects aged 35-80 years old for eight months. The impedance parameters used were PA ratios of 50 kHz and 100 kHz and PA ratios of 50 kHz and 200 kHz. Skeletal muscle PA showed a significant difference of approximately 3-4% between healthy subjects and patients with ALS and showed a change of approximately 4° of the progression of ALS in eight months. Rutkove et al. [9] evaluated the progress of stage and changes in skeletal muscle function with steroid therapy in healthy children and children with DMD aged 2-14 years by EIM. Two frequency phase ratios were used to determine the impedance parameters. Compared with healthy children, children with DMD showed a significant decrease with advancing disease stages. Meanwhile, in children treated with corticosteroids during follow-up, an improvement was observed in the phase ratio of the two frequencies. This change may not be a decrease in skeletal muscle fat or connective tissue but may be a sensitive indicator of the response of PA to inflammation in skeletal muscle and inhibition of muscle fiber destruction. However, no studies have been conducted on skeletal muscle disorders associated with acute inflammation after surgery using EIM. In this case since changes during the postoperative course, it is worthwhile to distinguish between PA and *β*, which constitute not only a single frequency but also a central frequency band.

To date, there is no established method to evaluate the quality of skeletal muscle during rehabilitation in the acute stage. Although ultrasonography is likely to be effective in assessing skeletal muscle function in severe disease, it is important to pay attention to errors due to these factors, as more sophisticated techniques and equipment are required for changes in intravascular and extravascular water balance and anatomical position associated with muscle atrophy. Meanwhile, the advantages of EIM are that it is not affected by dehydration [10] and that there is no need for advanced technology at the time of measurement. However, the EIM is affected by the electrodeposition and contact conditions due to impedance measurements. For an accurate evaluation, individual electrodes that are tailored to the thickness of the fat in the muscle of interest and the shape of the contact surface are desirable. Various handheld electrodes have been developed in previous studies [11,12]. However, many electrodes are directed to the muscles of the upper extremities with a small fat thickness, and it is necessary to develop electrodes that can be applied to the muscles of the lower extremities and obese patients.

Limitation

This case report has several limitations. First, qualitative changes in skeletal muscle were evaluated only using ultrasonography. Anatomical evidence of qualitative changes could not be provided because no invasive evaluation was performed. Second, follow-up was terminated on POD 14. Muscle strength and PA were reduced compared with preoperative values and discharged from the hospital, generally improved at 12 weeks [13], which may also prove the value of EIM by evaluating the course of improvement. The results of this case report also warrant further study on acute inflammation of the skeletal muscle and the superior results of the evaluation using EIM.

## Conclusions

This case report demonstrates the effectiveness of EIM evaluation for inflammatory changes in skeletal muscle function that occur after cardiac surgery. In the acute phase, skeletal muscle function is markedly impaired in a short period due to qualitative changes in skeletal muscle caused by inflammation. Although these standard evaluations do not exist, EIM allows objective measurement of inflammatory skeletal muscle conversion with multiple parameters. Further research is needed on the significance of each parameter and the accuracy of the numerical values.

## Appendices

Grip strength

The equipment used was a Takei T.K.K.5401 GRIP-D handgrip dynamometer (Takei Scientiﬁc Instruments Co., Ltd, Japan). The measurement was taken while the subject was standing, with the arm at the side and the elbow extension.

Leg extensor strength

The equipment used was a Strength Ergo 5 (BK-ERG-051, Mitsubishi Electric, Japan). The measurement was performed in isokinetic mode at a rotation speed of 50 rpm and five consecutive drives, and then the peak torque (Nm) was calculated.

Electrical impedance myography

A Bioelectrical Impedance Spectroscopy Unit (SFB7, Impedimed, Australia) was used. The electrodes used for measurement were plastic sprint materials (Easy Foam, Sakai Medical, Japan), and handheld electrodes with an arc-shaped electrode mounting surface were used. The electrode mounting surface was 80 mm (R 66 mm arc) × 130 mm wide, and the tape electrodes (Ag-AgCl, 10 mm × 80 mm) were mounted in a four-electrode arrangement. The distance between the potential electrodes was 100 mm, and the distance between the potential electrodes was 20 mm. A hard-type jelly (Conductor Transmission Gel, Chattanooga) was used during the measurement to avoid skin pressure and resistance. The rectus femoris muscle was measured from the anterior superior iliac spine to the midpoint of the upper patellar margin, Unified. For the analysis of impedance, parameters were calculated by arc optimization using the Cole-Cole model using 172 points of resistance and reactance with frequencies in the range of 10-300 kHz, and PA were calculated from the numerical values in the same manner as in a previous study [3]. The EIM was measured twice, and the average value was calculated.
